# A Garden Party at the Cancer Hospital

**Published:** 1888-09-15

**Authors:** 


### A Garden Party at the Cancer Hospital.

If there is one disease more than another which the outside world looks upon as incompatible with anything bright and festive it is cancer. That gnawing fox preying upon the most vital parts gives little respite to its victims; pain may be more or less acute, but pain is always present. Still, if the class of ailments generally classified under the title of cancer are as cruel and merciless as the legendary fox, many of those whom it attacks display the fortitude of the Spartan boy. Some there are among the patients in the Cancer Hospital at Brompton for whom all attempt at courage is past, who have no desire left for anything but death, or, till that comes, for some narcotic to dull their sufferings. But for many there is still hope of life and health regained, and others show that quiet, brave power of looking fate in the face which made the old Persian sage exclaim "Hope is a slave, despair is a free man."

The bondsmen of hope and the freedmen of despair were both represented among the seventy patients who gathered in the hospital garden on Wednesday, the 5th inst. Not a patient was left in the wards whom it would have been safe to trust outside, and the strong arms of nurses and attendants were put in requisition to convey those sufferers who were unable to walk. The strong arms of friends and husbands too, were permitted to give their kindly, though unskilled aid. "I did that nice, didn't I, Polly?" asked a stalwart costermonger of the pale, thin wife he had carried from ward to garden. "I don't think I 'urt you a bit." "No, Dick, not a bit," came the reply, with a faint loving smile, though those who had noticed the expression of Polly's drawn white face might justly have doubted if the assurance was true. But often the true wife would rather endure pain from the man she loves than kindness from strange hands, and it is hardly probable that Polly's falsehood remained more permanently recorded in the Angel's book of doom than Uncle Toby's famous solitary oath.

Everyone was determined to forget pain and anxiety for that afternoon at least. The patient who was to-day testing her strength to see if she was able to go home for a holiday, kept her mind fixed on the week or two she was to spend with her husband and children, and would not remember that after the holiday was past she would come back to the hospital again—to die. Those who were rejoicing in the society of friends and kindred forgot the long periods of separation that made this reunion seem the more delightful. And those whose friends were far away, and could not be with them at this annual festival, clung the more to the kind, familiar forms of the nurses, and were the most appreciative listeners to the conversation of the visitors, and paid the most attention to the band, and took their high tea with the most determined enjoyment. Meanwhile Mr. Hughes (the secretary) and the matron (Miss Rogers), with the chaplain and the nurses, went about among the patients and their friends, giving a kind word wherever they passed, and leaving a smile behind; and the afternoon sped happily on till six o'clock, when the band played the National Anthem, and the friends went away, leaving the patients to return to their wards, tired indeed, but cheered and brightened by this break in the quiet monotony of hospital life.

